# Observational study: Temporal dynamics of lipoprotein(a) after out-of-hospital cardiac arrest: The Biobank Working Group

**DOI:** 10.1016/j.resplu.2026.101449

**Published:** 2026-08-15

**Authors:** Daniel Kogler, Christian Schoergenhofer, Alexandra Lipa, Luca Kurz, Matthias Weiss-Tessbach, Ingrid Magnet, Michael Holzer, Michael Poppe, Teresa Lindmayr, Magdalena Boegl, Thomas Szekeres, Michael Schwameis, Juergen Grafeneder

**Affiliations:** a Department of Emergency Medicine, Medical University of Vienna, Austria; b Department of Clinical Pharmacology, Medical University of Vienna, Austria; c Department of Laboratory Medicine, Medical University of Vienna, Austria; d Clinical Division of Gynecological Endocrinology and Reproductive Medicine, Department of Obstetrics and Gynecology, Medical University of Vienna, Austria

**Keywords:** Resuscitation, Post resuscitation care, Neurological prognostication, Biomarkers

## Abstract

**Background:**

Out-of-hospital cardiac arrest (OHCA) is a major cause of death in the USA and Europe, with survival rates around 10%. Lipoprotein(a) [Lp(a)] is an established cardiovascular risk factor, but its temporal profile following OHCA is unknown.

**Methods:**

In this retrospective study at a tertiary cardiac arrest center, 50 adult patients with OHCA of presumed cardiac origin were included and stratified by 6-month neurological outcome (CPC 1–2 vs. CPC 3–5). Longitudinal Lp(a) concentrations were analyzed using a linear mixed-effects model with fixed effects for time point, neurological outcome, and their interaction. Exploratory logistic regression assessed associations between Lp(a) at ROSC and neurological outcome.

**Results:**

26 patients had a favorable outcome, and 22 had an unfavorable outcome (two patients were excluded after analysis due to problems with the sample). Baseline characteristics were similar, except for longer low-flow duration (*p* = 0.005), higher cummulative epinephrine dosage (*p* = 0.025) and lower pH (*p* = 0.013) in the unfavorable group. Absolute Lp(a) concentrations at ROSC did not differ between groups (*p* = 0.646) and were not associated with outcome. In the mixed-effects model, both timepoint (*p* < 0.0001) and the timepoint × outcome interaction (*p* = 0.020) were significantly associated with Lp(a) concentrations. Lp(a) remained relatively stable in patients with a favorable outcome, whereas it declined significantly over 48 h in those with an unfavorable outcome.

**Conclusion:**

Lp(a) exhibits a time-dependent trajectory after OHCA that differs by neurological outcome. While baseline levels are not predictive, patients with unfavorable outcomes show a significant decline over time, suggesting a dynamic response possibly linked to disease severity.

## Highlights

• Lipoprotein(a) exhibited a time-dependent trajectory after out-of-hospital cardiac arrest.
• Baseline Lipoprotein(a) at return of spontaneous circulation was not associated with 6-month neurological outcome.
• Lipoprotein(a) trajectories differed by outcome, with a significant decline observed in patients with unfavorable outcomes.

## Introduction

Out-of-hospital cardiac arrest (OHCA) is a major cause of death worldwide, with approximately 350,000 EMS-treated cases annually in Europe and 260,000 in the United States.^1^, ^2^ Only 10% of patients survive to hospital discharge, and up to 70% of patients admitted to hospital after cardiac arrest die from the effects of brain injury, even when initial resuscitation is successful.^1^, ^3^, ^4^

Neurological prognostication after OHCA remains difficult because no single parameter can accurately predict the outcome. As a result, current guidelines recommend a multimodal approach. To date, neuron-specific enolase (NSE) is the only biomarker advised for neurological prognostication,^1^, ^5^ with Neurofilament light being increasingly recognized.^6^ Identifying additional biomarkers that reflect the extent of post-arrest injury could help stratify patients earlier, guide targeted therapies, and tailor rehabilitation programs.

Lipoprotein(a) [Lp(a)] was first detected in 1963,^7^ and ever since, evidence has grown that Lp(a) plays a vital role in cardiovascular disease. Lp(a) is reported to be an independent risk factor and predictor for cardiovascular events.^8^ This includes the risk of myocardial infarction,^10^, ^11^, ^12^ the risk of cardiac mortality in patients with acute coronary syndromes,^13^ and the risk of sudden cardiac death.^9^, ^10^ Despite progress in prevention, cardiovascular disease continues to be the leading cause of OHCA.^11^, ^12^, ^13^

Post-cardiac arrest syndrome is driven by systemic ischemia–reperfusion injury and a profound inflammatory response, in which IL-6 plays a central role.^14^ Lp(a), whose hepatic production is directly upregulated by IL-6 via response elements in the *LPA* gene, is therefore hypothesized to undergo dynamic changes during this phase.^15^ Unlike isolated myocardial infarction, OHCA produces global ischemia–reperfusion, and the resulting Lp(a) kinetics remain uncharacterized. Understanding these temporal changes is clinically important for three reasons: (1) Lp(a) trajectory may reflect the severity of the post-arrest inflammatory response and thus carry prognostic value; (2) Lp(a)'s prothrombotic and proinflammatory properties — mediated through oxidized phospholipids, fibrinolysis inhibition, and platelet activation — may actively contribute to secondary injury^16^; and (3) knowledge of Lp(a) kinetics is important to determine the optimal timing for Lp(a) measurement in OHCA survivors for accurate long-term cardiovascular risk stratification.

This study therefore aimed to characterize the temporal kinetics of Lp(a) during the first 48 h after OHCA and to explore their association with neurological outcomes.

## Methods

This study analyzed data from the resuscitation database and biobank maintained at the Department of Emergency Medicine at the Medical University of Vienna. The Medical University of Vienna functions as a tertiary care center in central Vienna, serving a population of approximately 2 million residents. The Emergency Department is a certified cardiac arrest center and holds the gold certification as a center of excellence approved by the Extracorporeal Life Support Organization (ELSO), managing about 300 resuscitations each year, including 60 extracorporeal cardiopulmonary resuscitations (ECPR).^17^

Patients suffering from OHCA were prospectively included in this database. The database and its analysis received approval from the Ethics Committee of the Medical University of Vienna (1902/2017).

This exploratory, hypothesis-generating biomarker study was based on a predefined subset of 50 patients selected from a prospective registry of 1204 consecutive patients enrolled between October 2019 and November 2022. Eligible patients were aged ≥18 years, had experienced an out-of-hospital cardiac arrest of presumed cardiac origin, achieved sustained ROSC, and had serial plasma samples available at all predefined study time points. Patients were randomly selected within neurological outcome strata to obtain an approximately balanced distribution of favorable (CPC 1–2) and unfavorable neurological outcomes (CPC 3–5), thereby facilitating exploratory comparisons of temporal Lp(a) trajectories between outcome groups.

The sample size was determined pragmatically based on the availability of suitable serial biospecimens and the laboratory resources required for repeated Lp(a) measurements. Given the absence of prior data on serial Lp(a) trajectories after cardiac arrest and the exploratory, hypothesis-generating nature of this nested biomarker study, no formal a priori sample size calculation was performed; instead, the sample size was determined pragmatically based on the availability of serial biospecimens suitable for repeated Lp(a) measurements and available laboratory resources.

For this study, the neurological outcome at six months was considered the primary outcome, as recommended by the Core Outcome Set for Cardiac Arrest (COSCA) group. Blood samples were collected immediately after return of spontaneous circulation (ROSC) and then again at 24 and 48 h. Samples were stored at −80 °C until analysis. Lipoprotein(a) concentrations were measured using a particle-enhanced turbidimetric immunoassay (LPA2, Roche Diagnostics, Switzerland).

Patient characteristics are presented according to neurological outcome: CPC 1–2 and CPC 3–5. Continuous variables are expressed as median (IQR), categorical variables are shown as counts and percentages. Between-group comparisons were performed using the Mann-Whitney *U* test for continuous variables and the Chi-squared test for categorical variables.

Missing data were not imputed; all analyses were performed using the available observations. Lp(a) concentrations below the limit of detection (reported as <7.0 nmol l^−1^) were managed using a lower limit of detection (LOD/2) approach.

Longitudinal changes in Lp(a) levels were analyzed using linear mixed-effects models with patient ID included as a random intercept to account for repeated measurements and within-subject correlation. Fixed effects included timepoint, neurological outcome at 6 months, and their interaction. When an interaction was present, post hoc pairwise comparisons were performed using estimated marginal means with Bonferroni correction for multiple comparisons.

For exploratory purposes, we assessed the association between Lp(a) levels at ROSC and neurological outcome at 6 months using logistic regression. In the unadjusted model, Lp(a) was entered as the independent variable and the neurological outcome as the dependent variable. Because of the small sample size, we included only variables in the multivariable logistic regression model that were significantly different between outcome groups in the baseline group comparisons.

To assess whether Lp(a) risk categories changed over time (ROSC, 24 h, 48 h), Lp(a) levels were categorized according to European Atherosclerosis Society (EAS) guideline recommended risk thresholds into low (<75 nmol l^−1^), gray-zone (75–125 nmol l^−1^), and high (>125 nmol l^−1^) risk groups.^18^ We then fitted an ordinal mixed-effects logistic regression model with risk category as the dependent variable, time point as a fixed effect, and patient ID as a random intercept to account for repeated measurements.

For comparison with the established acute-phase parameter CRP, both Lp(a) and CRP were visualized over time using spaghetti plots, with median trajectories stratified by neurological outcome.

A two-sided *p*-value < 0.05 was considered statistically significant. Statistical analyses were performed using R (“Cucumberleaf Sunflower” Release, R Foundation for Statistical Computing, Vienna, Austria).

## Results

A total of 50 patients were classified according to their 6-month neurological outcome. Twenty-six patients had a favorable outcome (CPC 1–2), and 22 patients had an unfavorable outcome (CPC 3–5). Two patients were excluded due to missing samples or difficulties while handling the laboratory samples.

Baseline characteristics were similar between groups, with no significant differences in age, sex, height, weight, or BMI (all *p* > 0.05, Table 1). Resuscitation variables were mostly comparable, although low-flow time was significantly longer in the unfavorable group (40 min [22–54] vs. 15 min [6–25], *p* = 0.005). Initial pH was significantly lower in the unfavorable group (7.14 [7.08–7.23] vs. 7.25 [7.16–7.32]), *p* = 0.013). Median cumulative epinephrine dosage was higher in the unfavorable group (1 mg [0–2] vs. 2 mg [1–6], *p* = 0.025). NSE measurements were available in 3 of 47 patients (6.4%) at 24 h, 28 of 48 patients (58.3%) at 48 h, and 21 of 48 patients (43.8%) at 72 h. Given the substantial and time-dependent missingness, NSE was excluded from subsequent analyses.

**Table 1:** Patient characteristics stratified by 6-month neurological outcome.

**Variable**	**Favorable**	**Unfavorable**	***p*-value**	**Missing (%)**
*n*	26	22		

**Patient demographics**				
Age (median [IQR])	57.5 [48.3, 65.3]	61.5 [53.3, 69.5]	0.214	0
Male *n* (%)	16 (61.5)	17 (77.3)	0.390	0
Height (median [IQR])	177.0 [165.0, 185.0]	175.0 [170.5, 180.0]	0.708	0
Weight (median [IQR])	88.0 [62.5, 94.3]	87.5 [80.0, 100.0]	0.205	0
BMI (median [IQR])	25.44 [23.12, 30.28]	27.24 [26.12, 31.05]	0.086	0

**Resuscitation characteristics**				
No-flow (median [IQR])	1 [0, 1]	1 [1, 3]	0.249	8
Low-flow (median [IQR])	15 [6, 25]	40 [22, 54]	**0.005**	8
Witnessed arrest *n* (%)	24 (96.0)	16 (72.7)	0.060	0
Epinephrine (median [IQR])	1 [0, 2]	2 [1, 6]	**0.025**	0
No BLS *n* (%)	0	4 (100.0)	−	0
Shockable rhythm *n* (%)	25 (96.2)	18 (81.8)	0.252	0
ECPR *n* (%)	2 (7.7)	6 (27.3)	0.154	0

**Laboratory parameters**				
CRP (median [IQR])	0.17 [0.06, 0.36]	0.28 [0.10, 0.86]	0.141	0
LP(a) (median [IQR])	13.00 [7.00, 86.50]	21.50 [4.62, 152.50]	0.646	0
Lactate (median [IQR])	4.65 [3.92, 6.63]	6.50 [4.82, 9.78]	0.058	0
pH (median [IQR])	7.25 [7.16, 7.32]	7.14 [7.08, 7.23]	**0.013**	0

*n* indicates the number of patients per outcome group.

Favorable outcome (CPC 1–2), unfavorable outcome (CPC 3–5).

Between-group comparisons: Mann-Whitney *U* test for continuous variables; Chi-squared test for categorical variables.

Age years, height cm, weight kg, BMI kg m^−2^, no-flow minutes, low-flow minutes, cumulative epinephrine mg, Lp(a) at ROSC nmol l^−1^, C-reactive protein (CRP) at ROSC mg dl^−1^, Lactate mmol l^−1^.

Lp(a) concentrations at ROSC were measured at 13.00 nmol l^−1^ [7.00–86.50 nmol l^−1^] in the favorable group and 21.50 nmol l^−1^ [4.62–152.50 nmol l^−1^] in the unfavorable group (*p* = 0.646). CRP at ROSC was measured at 0.17 mg dL^−1^ [0.06–0.36 mg dL^−1^] in the favorable group and 0.28 mg dL^−1^ [0.11–0.86 mg dL^−1^] in the unfavorable group (*p* = 0.141).

Neurological outcomes comprised CPC 1 (47.9%), CPC 2 (6.3%), and CPC 5 (45.8%); no patients had CPC scores of 3 or 4. Among patients with CPC 5, withdrawal of life-sustaining therapy (WLST) occurred in 68.2%, most commonly because of hypoxic–ischemic brain injury (50.0%). Other causes of death included multiorgan failure (27.3%), bleeding (4.5%), persistent shock (4.5%), pulmonary causes (4.5%), and unknown causes (9.1%).

A total of 8 patients received ECPR – two patients (7.7%) in the favorable group and six (27.3%) patients in the unfavorable group. Basic life support (BLS) was attempted in all patients with a favorable outcome, whereas four patients in the unfavorable group did not receive BLS.

**Fig. 1:**
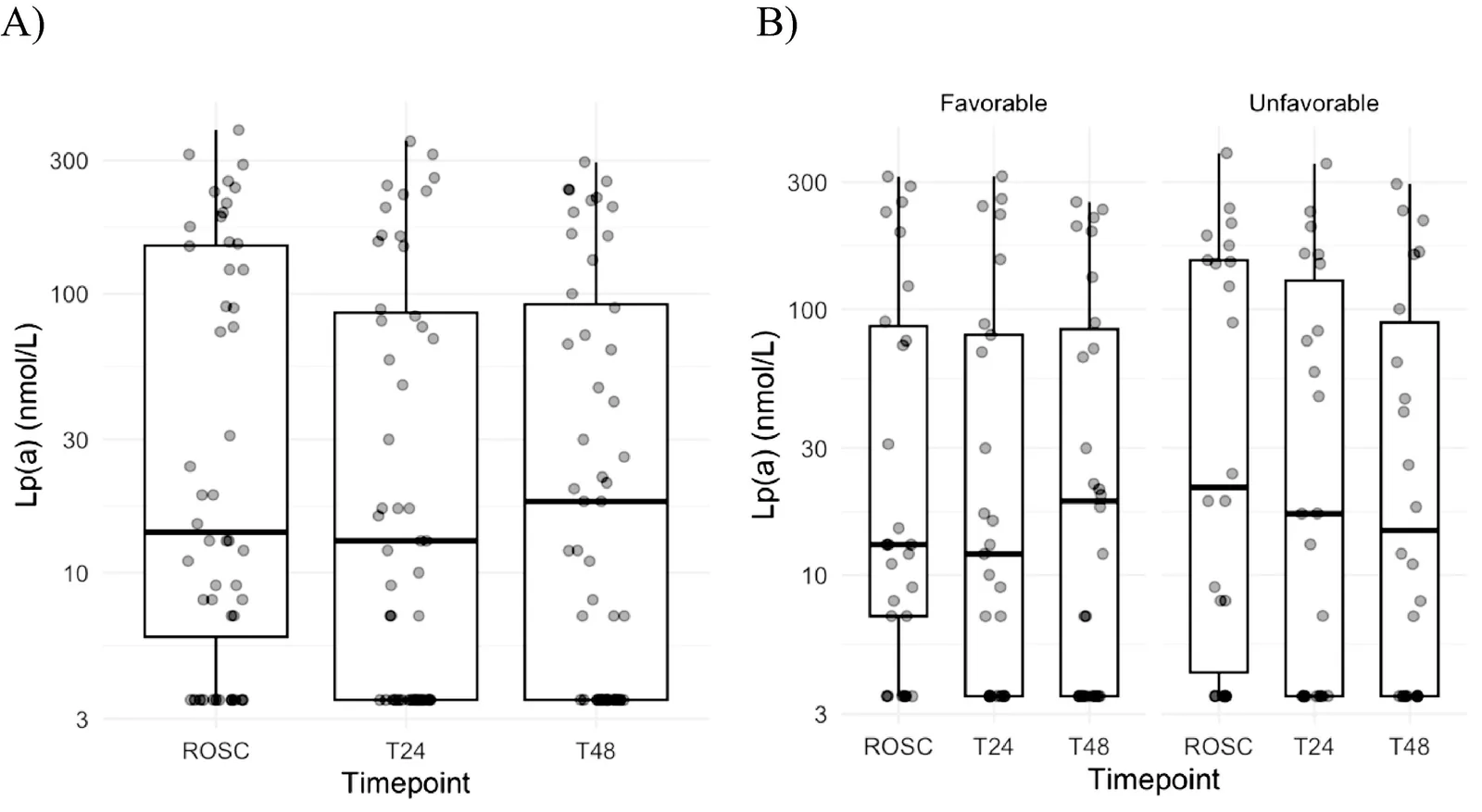
**Distribution of plasma lipoprotein(a) [Lp(a)] concentrations over time**. **(A)** Boxplots showing Lp(a) concentrations at return of spontaneous circulation (ROSC), 24 h, and 48 h in the overall study cohort (*n* = 48). **(B)** Boxplots showing Lp(a) concentrations at ROSC, 24 h, and 48 h stratified by 6-month neurological outcome (favorable: CPC 1–2, *n* = 26; unfavorable: CPC 5, *n* = 22). Boxes represent the interquartile range, the horizontal line indicates the median, whiskers extend to 1.5× the interquartile range, and individual observations are shown as dots. The *y*-axis is displayed on a logarithmic scale in both panels. ROSC, return of spontaneous circulation; CPC, Cerebral Performance Category.

Lp(a) concentrations remained relatively stable over time in patients with a favorable neurological outcome, whereas they declined over time in patients with an unfavorable neurological outcome, consistent with the significant time-by-outcome interaction (Fig. 1).

In the linear mixed-effect model, both timepoint (*p* < 0.0001) and the interaction between timepoint and neurological outcome (*p* = 0.020) were significantly associated with Lp(a) concentrations, whereas neurological outcome alone was not (*p* = 0.7445).

Post-hoc comparisons with Bonferroni correction showed no significant changes in Lp(a) concentrations over time in patients with a favorable neurological outcome. In contrast, patients with unfavorable outcome demonstrated a significant decline in Lp(a) concentrations, with higher levels at ROSC compared with 24 h (*p* = 0.006) and 48 h (*p* ≤ 0.001). No significant difference was observed between 24 h and 48 h. No significant differences in absolute Lp(a) levels were detected between outcome groups at any individual time point.

A sensitivity analysis excluding all patients treated with ECPR yielded similar temporal Lp(a) trajectories, with both the effect of time (*p* = 0.0003) and the interaction between time and neurological outcome (*p* = 0.0303) remaining statistically significant (Supplementary Fig. S2).

**Fig. 2:**
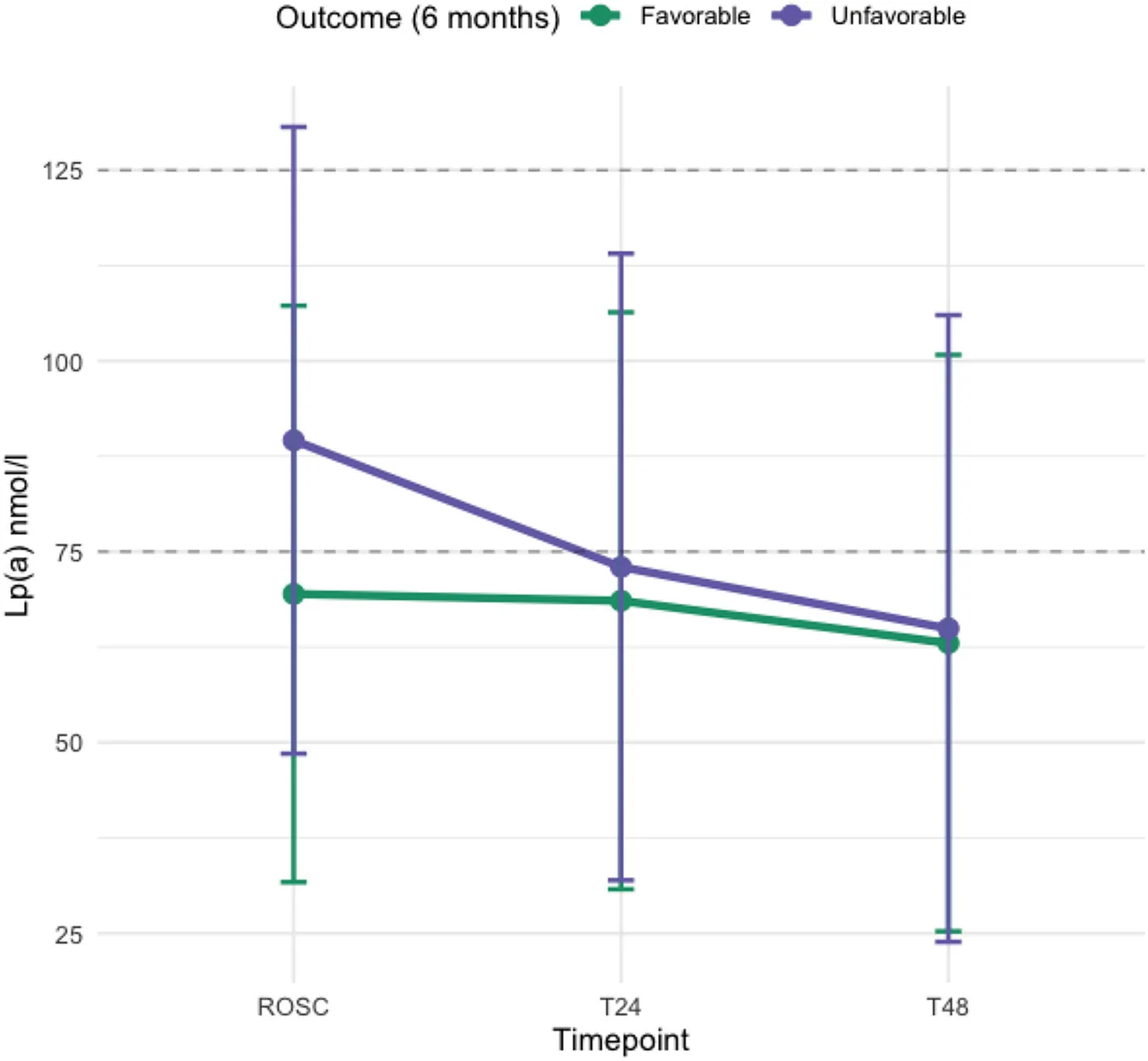
**Estimated marginal means of Lp(a) concentrations at return of spontaneous circulation (ROSC), 24 h, and 48 h, stratified by 6-month neurological outcome. Points represent estimated marginal means derived from a linear mixed-effects model including time point, neurological outcome, and their interaction as fixed effects, with patient ID as a random intercept. Error bars represent 95% confidence intervals. The analysis included 48 patients. Dotted horizontal lines indicate the European Atherosclerosis Society Lp(a) risk thresholds (<75 nmol/L for low risk and >125 nmol/L for high risk)**.

Lp(a) concentrations declined over time in both groups, although the decline was substantially more pronounced in patients with an unfavorable neurological outcome (Fig. 2, Fig. S1b).

In the univariable logistic regression, Lp(a) levels at ROSC were not associated with 6-month neurological outcome (*p* = 0.493). After adjustment for low-flow time, Lp(a) remained not associated with outcome (*p* = 0.092). In contrast, longer low-flow time was independently associated with an unfavorable neurological outcome (adjusted OR 1.06 per minute, 95% CI 1.02–1.12, *p* = 0.006).

The distribution of patients across EAS Lp(a) risk categories changed over time, with a decrease in the proportion classified as high risk (37.5% at ROSC vs. 27.1% at 48 h) and a corresponding increase in the intermediate-risk category, while approximately 60% remained in the low-risk category throughout (Table 2).

**Table 2:** Distribution of patients across European Atherosclerosis Society Lp(a) risk categories at each time point. Low Risk (<75 nmol/L), Gray-Zone (75–125 nmol/L) and High Risk (>125 nmol/L). Data are presented as *n* (%).

**Timepoint**	**Low risk**	**Gray-zone**	**High risk**
ROSC	28 (58.3)	2 (4.2)	18 (37.5)
24 h	28 (59.6)	4 (8.5)	15 (31.9)
48 h	29 (60.4)	6 (12.5)	13 (27.1)

Lp(a) categories based on EAS-recommended thresholds (<75, 75–125, and >125 nmol/L) tended to shift towards lower categories over time. In an ordinal mixed-effects logistic regression model, the odds of being in a higher Lp(a) category were lower at 24 h than at ROSC, although this difference was not statistically significant (OR 0.305, 95% CI 0.035–2.63; *p* = 0.280). At 48 h, the odds of being in a higher category were significantly lower than at ROSC (OR 0.032, 95% CI 0.002–0.61; *p* = 0.022) (Table 3).

**Table 3:** Ordinal mixed-effects logistic regression model for Lp(a) risk groups over time.

**Variable**	**Odds ratio**	**95% CI**	***p*-value**
24 h vs ROSC	0.305	0.035–2.63	0.280
48 h vs ROSC	0.032	0.002–0.61	**0.022**

**Fig. 3:**
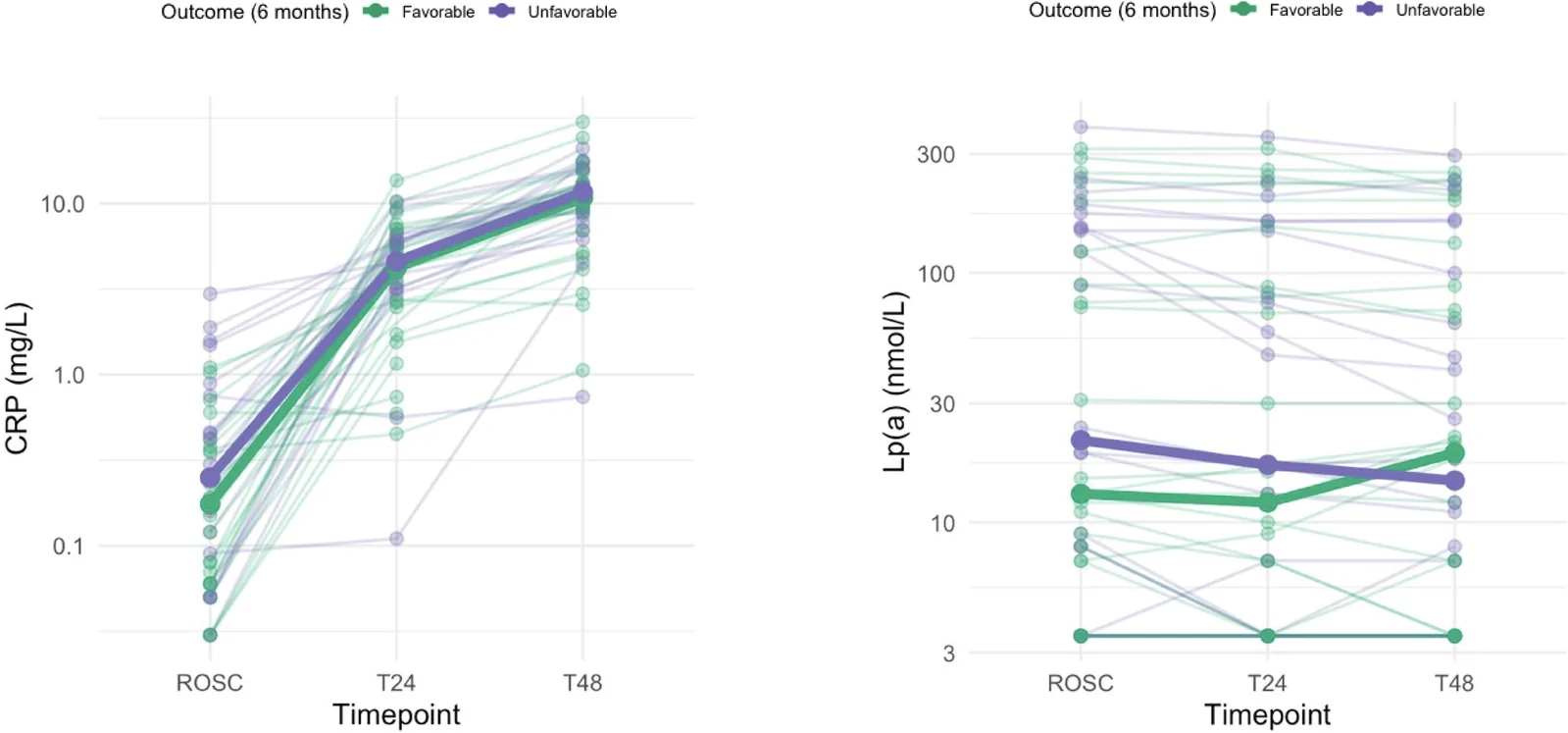
**Individual trajectories of C-reactive protein (CRP) and lipoprotein(a) [Lp(a)] concentrations from return of spontaneous circulation (ROSC) to 24 and 48 h, stratified by 6-month neurological outcome. Favorable neurological outcome was defined as Cerebral Performance Category (CPC) 1–2 and unfavorable outcome as CPC 3–5. Thin lines represent individual patients, and bold lines represent group median trajectories. Both *y*-axes are displayed on a logarithmic scale. The analyses included 47 patients for CRP and 48 patients for Lp(a). Data are presented descriptively; no statistical comparisons are shown in this figure**.

Individual trajectories of CRP and Lp(a) concentrations are shown in Fig. 3. CRP concentrations increased markedly between ROSC and 48 h in both neurological outcome groups. In contrast, Lp(a) concentrations showed only modest temporal changes but remained consistently higher in patients with unfavorable neurological outcomes. While substantial interindividual variability was observed, the descriptive trajectories were consistent with the mixed-effects model results.

## Discussion

In this study, the temporal trajectory of Lp(a) within the first 48 h after ROSC differed between outcome groups, while absolute Lp(a) levels did not significantly differ between groups at any individual time point. Importantly, the observed temporal Lp(a) pattern remained similar after exclusion of all patients treated with ECPR, suggesting that the main findings were not driven by this clinically distinct subgroup.

Lp(a) is a known risk factor for cardiovascular disease. In previous studies, an Lp(a) concentration above 105 nmol/l has been established as a cardiovascular risk factor.^14^, ^15^ Lp(a) values above 72 nmol l^−1^ have even been shown to be an independent risk factor for cardiac mortality in patients after myocardial infarction.^19^ Yet, in this population, where all OHCAs were due to presumed cardiac cause, even though median values and the upper ranges were numerically higher in patients with unfavorable outcomes, distributions largely overlapped. Given this observation, no significant association between Lp(a) and neurological outcome was found in either crude or multivariable regression analyses. This lack of association may partly reflect limited statistical power, given the interindividual variability in Lp(a) concentrations and the modest sample size.

Lp(a) concentrations are largely (80–90%) genetically determined^18^, ^20^ and concentration is generally stable over time.^21^ However, Lp(a) shows a markedly different profile in acute settings.

A study by Ziogos et al. showed that Lp(a) concentration is higher 6 months after myocardial infarction compared to a baseline Lp(a) taken within 24 h after admission.^22^ Other studies have shown that in the acute phase of acute coronary syndrome, Lp(a) concentrations initially decrease within the first 12 h before returning to baseline. A greater decrease in this time frame was independently associated with a major adverse cardiovascular event during a three-year follow-up period.^23^ Interestingly, similar to our study, in patients with sepsis or severe burn injuries, Lp(a) exhibits characteristics of a negative acute-phase reactant, with concentrations decreasing rapidly and transiently during the acute phase.^24^

In this context, we observed an inverse temporal pattern between Lp(a) and CRP, with rising CRP levels and a concomitant decline in Lp(a), suggesting divergent biomarker kinetics during the early post-ROSC phase. These findings may reflect context-dependent alterations in lipoprotein hemostasis during the post-arrest inflammatory response.

Studies show that a single measurement Lp(a) is generally sufficient to assess cardiovascular risk, as Lp(a) levels are considered stable over time. This assumption particularly applies to individuals in the low- and high-risk categories. Only in patients within the intermediate (“gray zone”) range may repeated measurements be considered, as values in this group can shift toward either lower- or higher-risk categories.^21^, ^25^

In our study, however, the odds of being classified into a higher Lp(a) risk category decreased significantly within the first 48 h, which may suggest a potential consumption or dynamic regulation of Lp(a) in this acute setting.

The post-cardiac arrest syndrome has been named a “sepsis-like” syndrome, which includes systemic ischemia–reperfusion injury, brain damage, and inflammatory responses (4). These processes may also affect the temporal dynamics of Lp(a), following OHCA, and could partly explain the changes in Lp(a) concentrations observed in the early post-resuscitation period. Endothelial injury plays a major role in the post-cardiac arrest syndrome.^26^, ^27^, ^28^ Previous studies have demonstrated a close relationship between Lp(a) and inflammatory processes, with Lp(a) associated with systemic inflammation and capable of negatively impacting the endothelium.^29^ This could explain the different trajectory in patients with an unfavorable neurological outcome, possibly linked to disease severity.

Another key finding of this study is the distinct temporal pattern of Lp(a) in patients with an unfavorable neurological outcome, potentially reflecting the underlying pathophysiological processes described above. Although absolute Lp(a) concentrations were not associated with outcome, their temporal dynamics may offer additional prognostic value. The potential predictive value of declining Lp(a) concentrations should be investigated in future studies with larger samples.

Several factors may have influenced the observed Lp(a) kinetics. First, acute myocardial infarction—the leading cause of OHCA—is itself associated with dynamic changes in Lp(a), with concentrations initially decreasing before rising over subsequent days, although the trajectory observed after global ischemia–reperfusion may differ from that following isolated myocardial infarction.^14^, ^30^ Second, post-cardiac arrest syndrome is characterized by a profound IL-6–mediated inflammatory response.^31^, ^32^ Because IL-6 directly upregulates hepatic LPA gene expression, sustained inflammation may influence circulating Lp(a) concentrations after resuscitation.^33^ At the same time, transient hepatic dysfunction and severe systemic inflammation during the early post-arrest phase may suppress Lp(a) synthesis,^24^ resulting in competing biological mechanisms that could contribute to the observed temporal kinetics. Finally, renal dysfunction and lipid-lowering therapies may also influence circulating Lp(a) concentrations.^34^, ^35^ However, statins have little effect on Lp(a), PCSK9 inhibitor use was uncommon in our cohort, and our sample size did not permit adequately powered multivariable analyses to adjust for all potential confounders.

### Limitations

This study is exploratory and observational, with a relatively small sample size from a single center. Potential confounding factors inherent to post-arrest care may have influenced Lp(a) levels, which were measured only during the first 48 h.

Because patients were randomly selected within predefined neurological outcome strata rather than sampled from the registry as a whole, the study cohort was not intended to be representative of the overall cardiac arrest population. This enriched sampling strategy may have introduced selection bias and led to overly optimistic estimates of biomarker performance. The study was not formally powered to detect predefined effect sizes. Accordingly, the observed prognostic performance should be considered exploratory, and both the biomarker findings and between-group comparisons require validation in larger, unselected prospective cohorts.

Neurological and inflammatory biomarkers were not prospectively and systematically collected. Although limited NSE measurements were available as part of routine clinical care, the substantial and likely non-random missingness precluded meaningful analyses. Neurofilament light chain was not available in this cohort.

Furthermore, patients undergoing extracorporeal cardiopulmonary resuscitation (ECPR) were included. Although ECPR is performed in only a minority of resuscitation cases, these patients may have a clinical course distinct from that of conventionally resuscitated patients, which may limit the generalizability of our findings.

Finally, the observed Lp(a) kinetics likely reflect the combined effects of post-resuscitation inflammation, ischemia–reperfusion injury, and patient-specific factors, including acute myocardial infarction, renal dysfunction, and lipid-lowering therapy. Because the present study was exploratory and not powered for comprehensive multivariable adjustment, residual confounding cannot be ruled out.

## Conclusions

In this study, Lp(a) showed a time-dependent trajectory following resuscitation from cardiac arrest, with a significant interaction between time point and neurological outcome. Absolute Lp(a) levels were not associated with 6-month neurological outcome. However, longitudinal analysis revealed distinct temporal patterns between outcome groups, with patients with unfavorable outcomes showing a significant decline in Lp(a) levels over the first 48 h. Additional research is needed to clarify the biological and clinical relevance of the observations.

## Author contributions

**D.K.** gathered and curated the data, developed the methodology, performed the analysis and visualization, interpretation of the results and wrote the original draft.

**J.G.** had the original idea, contributed to methodology, conceptualization, interpretation of the results and critical writing of the manuscript.

**C.S.** supervised the project, contributed to interpretated of the results and critical writing of the manuscript.

**A.L.** contributed to interpretation of the results and critical writing of the manuscript.

**L.K.** contributed to data curation, interpretation of the results and critical writing of the manuscript.

**M.W-T.** did the lab parameter testing, contributed to interpretation of the results and critical writing of the manuscript.

**M.S.** supervised the project, contributed to interpretation of the results and critical writing of the manuscript.

**T.L.** contributed to data curation, interpretation of the results and critical writing of the manuscript.

**T.S.** contributed to data curation, interpretation of the results and critical writing of the manuscript.

**I.M.** contributed to data curation, interpretation of the results and critical writing of the manuscript.

**M.P.** contributed to data curation, interpretation of the results and critical writing of the manuscript.

**M.H.** contributed to data curation, interpretation of the results and critical writing of the manuscript.

**M.B.** contributed to data curation, interpretation of the results and critical writing of the manuscript.

All named authors meet all four criteria for (co)-authorship provided by the Good Scientific Practice guidelines of the Medical University of Vienna. All authors read and approved the final manuscript.

## CRediT authorship contribution statement

**Daniel Kogler:** Writing – original draft, Visualization, Methodology, Formal analysis, Data curation. **Christian Schoergenhofer:** Writing – review & editing, Supervision, Methodology. **Alexandra Lipa:** Writing – review & editing. **Luca Kurz:** Writing – review & editing, Data curation. **Matthias Weiss-Tessbach:** Writing – review & editing, Data curation. **Ingrid Magnet:** Writing – review & editing. **Michael Holzer:** Writing – review & editing. **Michael Poppe:** Writing – review & editing. **Teresa Lindmayr:** Writing – review & editing. **Magdalena Boegl:** Writing – review & editing. **Thomas Szekeres:** Writing – review & editing. **Michael Schwameis:** Writing – review & editing, Supervision, Conceptualization. **Juergen Grafeneder:** Writing – review & editing, Visualization, Methodology, Conceptualization.

## Funding

None.

## Statement on the use of artificial intelligence

Artificial intelligence (AI) was used under careful supervision and validation. Open Evidence was used for literature search and ChatGPT (GPT-5.2) assisted in R programming and linguistic improvement. It is important to highlight that AI was not used for critical reasoning. The authors take full responsibility for this manuscript.

## Declaration of competing interest

The authors declare that they have no known competing financial interests or personal relationships that could have appeared to influence the work reported in this paper.

## Appendix A Supplementary material

Supplementary Data 1 Supplementary figures showing Lp(a) trajectories using a logarithmic scale and sensitivity analysis excluding patients treated with ECPR.

## References

[1] Del Rios M., Bartos J.A., Panchal A.R., Atkins D.L., Cabañas J.G., Cao D. (2025). Part 1: executive summary: 2025 American Heart Association guidelines for cardiopulmonary resuscitation and emergency cardiovascular care. Circulation.

[2] Empana J.-P., Lerner I., Valentin E., Folke F., Böttiger B., Gislason G. (2022). Incidence of sudden cardiac death in the European Union. J Am Coll Cardiol.

[3] Gräsner J.-T., Wnent J., Lefering R., Herlitz J., Masterson S., Maurer H. (2026). European registry of cardiac arrest study THREE (EuReCa- THREE) – EMS response time influence on outcome in Europe. Resuscitation.

[4] Perkins G.D., Callaway C.W., Haywood K., Neumar R.W., Lilja G., Rowland M.J. (2021). Brain injury after cardiac arrest. Lancet.

[5] Greif R., Lauridsen K.G., Djärv T., Ek J.E., Monnelly V., Monsieurs K.G. (2025). European Resuscitation Council guidelines 2025 executive summary. Resuscitation.

[6] Hoiland R.L., Rikhraj K.J.K., Thiara S., Fordyce C., Kramer A.H., Skrifvars M.B. (2022). Neurologic prognostication after cardiac arrest using brain biomarkers. JAMA Neurol.

[7] Berg K. (1963). A new serum type system in man—the L_p_ system. Acta Pathol Microbiol Scand.

[8] Kronenberg F., Utermann G. (2013). Lipoprotein(a): resurrected by genetics. J Intern Med.

[9] Kunutsor S.K., Khan H., Nyyssönen K., Laukkanen J.A. (2016). Lipoprotein(a) and risk of sudden cardiac death in middle-aged finnish men: a new prospective cohort study. Int J Cardiol.

[10] Li Y., Hong W., Li Y., Yu Q., Wang L., Jiang L. (2026). Predictive value of lipoprotein(a) and LPA genetic risk score for incident of sudden cardiac death. Mayo Clin Proc.

[11] Hawkes C., Booth S., Ji C., Brace-McDonnell S.J., Whittington A., Mapstone J. (2017). Epidemiology and outcomes from out-of-hospital cardiac arrests in England. Resuscitation.

[12] Mueller M., Grafeneder J., Schoergenhofer C., Schwameis M., Schriefl C., Poppe M. (2021). Initial blood pH, lactate and base deficit add no value to peri-arrest factors in prognostication of neurological outcome after out-of-hospital cardiac arrest. Front Med (Lausanne).

[13] Myat A., Song K.-J., Rea T. (2018). Out-of-hospital cardiac arrest: current concepts. Lancet.

[14] Nordestgaard B.G., Langsted A. (2024). Lipoprotein(a) and cardiovascular disease. Lancet.

[15] Tsimikas S., Marcovina S.M. (2022). Ancestry, lipoprotein(a), and cardiovascular risk thresholds. J Am Coll Cardiol.

[16] Kaiser Y., Daghem M., Tzolos E., Meah M.N., Doris M.K., Moss A.J. (2022). Association of lipoprotein(a) with atherosclerotic plaque progression. J Am Coll Cardiol.

[17] Magnet I., Behringer W., Eibensteiner F., Ettl F., Grafeneder J., Heinz G. (2025). Extracorporeal cardiopulmonary resuscitation: outcomes improve with center experience. Ann Emerg Med.

[18] Kronenberg F., Mora S., Stroes E.S.G., Ference B.A., Arsenault B.J., Berglund L. (2022). Lipoprotein(a) in atherosclerotic cardiovascular disease and aortic stenosis: a European Atherosclerosis Society consensus statement. Eur Heart J.

[19] Stubbs P. (1998). Lipoprotein(a) as a risk predictor for cardiac mortality in patients with acute coronary syndromes. Eur Heart J.

[20] Schmidt K., Noureen A., Kronenberg F., Utermann G. (2016). Structure, function, and genetics of lipoprotein(a). J Lipid Res.

[21] Awad K., Mahmoud A.K., Abbas M.T., Alsidawi S., Ayoub C., Arsanjani R. (2025). Intra-individual variability in lipoprotein(a) levels: findings from a large academic health system population. Eur J Prev Cardiol.

[22] Ziogos E., Vavuranakis M.A., Harb T., Foran P.L., Blaha M.J., Jones S.R. (2023). Lipoprotein(a) concentrations in acute myocardial infarction patients are not indicative of levels at six month follow-up. Eur Heart J Open.

[23] Saeki Y., Sawaguchi J., Akita S., Takamura T.-A., Fujibayashi K., Wakasa M. (2024). Initial decrease in the lipoprotein(a) level is a novel prognostic biomarker in patients with acute coronary syndrome. World J Cardiol.

[24] Mooser V., Berger M.M., Tappy L., Cayeux C., Marcovina S.M., Darioli R. (2000). Major reduction in plasma Lp(a) levels during sepsis and burns. Arterioscler Thromb Vasc Biol.

[25] Sung D.-E., Lee M.-Y., Kwon M.-J., Sung K.-C. (2025). Longitudinal changes and borderline reclassification of lipoprotein(a) compared with conventional lipids in over 230,000 adults. Atherosclerosis.

[26] Adams J.A. (2006). Endothelium and cardiopulmonary resuscitation. Crit Care Med.

[27] Caceres M.J., Schleien C.L., Kuluz J.W., Gelman B., Dietrich W.D. (1995). Early endothelial damage and leukocyte accumulation in piglet brains following cardiac arrest. Acta Neuropathol.

[28] Fink K., Schwarz M., Feldbrügge L., Sunkomat J.N., Schwab T., Bourgeois N. (2010). Severe endothelial injury and subsequent repair in patients after successful cardiopulmonary resuscitation. Crit Care.

[29] Pirro M., Bianconi V., Paciullo F., Mannarino M.R., Bagaglia F., Sahebkar A. (2017). Lipoprotein(a) and inflammation: a dangerous duet leading to endothelial loss of integrity. Pharmacol Res.

[30] Vavuranakis M.A., Jones S.R., Ziogos E., Blaha M.J., Williams M.S., Foran P. (2022). The trajectory of lipoprotein(a) during the peri- and early postinfarction period and the impact of proprotein convertase subtilisin/kexin type 9 inhibition. Am J Cardiol.

[31] Bro-Jeppesen J., Kjaergaard J., Stammet P., Wise M.P., Hovdenes J., Åneman A. (2016). Predictive value of interleukin-6 in post-cardiac arrest patients treated with targeted temperature management at 33 °C or 36 °C. Resuscitation.

[32] Bro-Jeppesen J., Kjaergaard J., Wanscher M., Nielsen N., Friberg H., Bjerre M. (2015). Systemic inflammatory response and potential prognostic implications after out-of-hospital cardiac arrest. Crit Care Med.

[33] Müller N., Schulte D.M., Türk K., Freitag-Wolf S., Hampe J., Zeuner R. (2015). IL-6 blockade by monoclonal antibodies inhibits apolipoprotein(a) expression and lipoprotein(a) synthesis in humans. J Lipid Res.

[34] Blumenthal R.S., Morris P.B., Gaudino M., Johnson H.M., Anderson T.S., Bittner V.A. (2026). 2026 ACC/AHA/AACVPR/ABC/ACPM/ADA/AGS/APhA/ASPC/NLA/PCNA guideline on the management of dyslipidemia. JACC.

[35] Tsimikas S., Fazio S., Ferdinand K.C., Ginsberg H.N., Koschinsky M.L., Marcovina S.M. (2018). NHLBI working group recommendations to reduce lipoprotein(a)-mediated risk of cardiovascular disease and aortic stenosis. J Am Coll Cardiol.

