# Supplementary material for: Observational study: Temporal dynamics of lipoprotein(a) after out-of-hospital cardiac arrest: The Biobank Working Group
Source: Resusc Plus. 2026 Aug 15;31:101449. doi: 10.1016/j.resplu.2026.101449 (PMC13543912; doi:10.1016/j.resplu.2026.101449)
Supplement: Supplementary file 1 [file mmc1.docx]

## Supplementary Material

**Figure S1a.** Estimated Marginal Means for Lp(a) at different timepoints (ROSC, 24 hours, 48 hours). The y-axis is displayed on a **linear scale**.

**Figure S1b.** Estimated Marginal Means for Lp(a) at different timepoints (ROSC, 24 hours, 48 hours). The y-axis is displayed on a **logarithmic scale**.

**Figure S2.**

Temporal trajectory of plasma lipoprotein(a) concentrations after exclusion of patients treated with extracorporeal cardiopulmonary resuscitation (ECPR), n=40. Estimated marginal mean Lp(a) concentrations are shown according to neurological outcome (favorable, CPC 1–2; unfavorable, CPC 5) at different timepoints (ROSC, 24 hours, 48 hours). Error bars indicate 95% confidence intervals, dotted lines represent the EAS threshold lines < 75 low risk, > 125 high risk.
